# Systems modelling as an approach for eliciting the mechanisms for hip fracture recovery among older adults in a participatory stakeholder engagement setting

**DOI:** 10.3389/fresc.2023.1184484

**Published:** 2023-06-23

**Authors:** John Pastor Ansah, Aloysius Wei-Yan Chia, Vanessa Jean Wen Koh, Wei Xuan Lai, Joyce Suang Bee Koh, Kiat Sern Goh, William Yeo, Tet Sen Howe, Dennis Chuen Chai Seow, Kaysar Mamun, Diraviyam Balasubramanian, Surendra Doraiswamy Varman, Andy Kuei Siong Yeo, Amal Elamin, Angelique Wei-Ming Chan, David Bruce Matchar

**Affiliations:** ^1^ Center for Community Health Integration, School of Medicine, Case Western Reserve University, Cleveland, United States; ^2^ Programme in Health Services and Systems Research, Duke-NUS Medical School, National University of Singapore, Singapore, Singapore; ^3^ Centre for Ageing Research and Education (CARE), Duke-NUS Medical School, Singapore, Singapore; ^4^ Department of Orthopaedic Surgery, Singapore General Hospital, Singapore, Singapore; ^5^ Department of Geriatric Medicine, Changi General Hospital, Singapore, Singapore; ^6^ Orthopaedic Diagnostics Centre, Singapore General Hospital, Singapore, Singapore; ^7^ Department of Geriatric Medicine, Singapore General Hospital, Singapore, Singapore; ^8^ Department of Orthopaedic Surgery, Changi General Hospital, Singapore, Singapore; ^9^ School of Human Sciences, Faculty of Education, Health and Human Sciences, University of Greenwich, London, United Kingdom

**Keywords:** hip fracture recovery, rehabilitation, resilience building, system dynamics modelling, group model building

## Abstract

**Introduction:**

Due to an aging population, the rising prevalence and incidence of hip fractures and the associated health and economic burden present a challenge to healthcare systems worldwide. Studies have shown that a complex interplay of physiological, psychological, and social factors often affects the recovery trajectories of older adults with hip fractures, often complicating the recovery process.

**Methods:**

This research aims to actively engage stakeholders (including doctors, physiotherapists, hip fracture patients, and caregivers) using the systems modeling methodology of Group Model Building (GMB) to elicit the factors that promote or inhibit hip fracture recovery, incorporating a feedback perspective to inform system-wide interventions. Hip fracture stakeholder engagement was facilitated through the Group Model Building approach in a two-half-day workshop of 25 stakeholders. This approach combined different techniques to develop a comprehensive qualitative whole-system view model of the factors that promote or inhibit hip fracture recovery.

**Results:**

A conceptual, qualitative model of the dynamics of hip fracture recovery was developed that draws on stakeholders' personal experiences through a moderated interaction. Stakeholders identified four domains (i.e., expectation formation, rehabilitation, affordability/availability, and resilience building) that play a significant role in the hip fracture recovery journey..

**Discussion:**

The insight that recovery of loss of function due to hip fracture is attributed to (a) the recognition of a gap between pre-fracture physical function and current physical function; and (b) the marshaling of psychological resilience to respond promptly to a physical functional loss via uptake of rehabilitation services is supported by findings and has several policy implications.

## 1. Introduction

With its high and increasing life expectancy (1), Singapore is likely to see a rise in osteoporosis, accidental falls, and consequent hip fractures (2, 3). Osteoporosis, a condition of progressive bone loss, resulted in an estimated 1.26 million hip fractures globally in 1990; this number is projected to increase to 4.5 million by 2050 (4), with the majority of this increase coming from Asia (4, 5). With a rising prevalence of osteoporosis, there are increasing concerns about hip fractures among older adults, as osteoporosis is associated with a six to seven-fold increase in the likelihood of hip fractures (6). In 2015, approximately 2,000 older adults were hospitalized locally due to hip fractures (7). Between 2007 and 2009, the age-standardized incidence of hip fractures in Singapore was estimated to be (per 100,000) 156 in men and 331 in women (8). Hip fractures are costly and demand considerable resources from the healthcare system, accounting for a large proportion of fracture-related healthcare expenditure and mortality in men and women over 50 (9–11). Considering this demographic reality, the economic burden of hip fractures on health systems is expected to increase. In Singapore, the mean cost of hospitalization due to hip fractures was approximately S$13,314 per patient in 2011, and an additional cost of S$2,690 may result from complications (12). Furthermore, considering post-acute rehabilitation, nursing, and caregiving costs (both formal and informal), total lifetime costs are likely to be considerably higher. In the United States, for instance, overall lifetime costs (taking into account lost productivity and other indirect costs) due to hip fractures were estimated to amount to more than US$81,000 (in 1997 US dollars) (13).

Furthermore, though hip fracture patients often undergo early surgical fixation, many cannot return to their pre-fracture levels of physical function and independence (14). Prioritizing only functional and performance targets alone may lead to poor outcomes for patients as it potentially excludes the preferences and psychosocial needs of patients recovering from hip fractures (15). Additionally, care transitions are a particularly vulnerable time for patients as they transition from the hospital post-surgery back into their home environment. As many older adult hip fracture patients often have other complicated comorbidities, these patients may have to transition between settings such as tertiary hospitals and community hospitals before discharging back into the community (16). During this transition process, patients typically receive fragmented information from various healthcare providers and even fragmented care, leading to unmet patient needs (16, 17). Should care transitions be poorly managed, this can result in hospital readmissions, poor functional outcomes, quality of life, and patient satisfaction (18, 19). During this time, caregivers, often family members, take on the overwhelming responsibility of caring for hip fracture patients. With the importance of caregivers in the recovery process, it is crucial to involve caregivers from the onset of care. Hence, it is essential to consider integrating patient - and family-centered care for hip fracture patients. Encouraging patients and their families to be involved in recovery can promote active collaboration and shared decision-making between patients, families, and clinicians (20). The benefits of this approach to care promote autonomy and independent living among hip fracture patients and decrease dependency on healthcare providers.

As alluded to above, the greater demand for care for hip fracture patients can strain informal caregivers, who are the primary source of help for dependent older adults in Singapore (21). As hip fracture patients have limitations in independent ambulation and functional ability, they are highly reliant on their caregivers in the performance of their daily activities, especially in the early stages of recovery and care transitions. Due to its acute nature, many informal caregivers assume the caregiver role with little or no preparation. Caregiving often involves difficult tasks and a complex relationship with the care recipient; this can affect informal family caregivers’ psychological and physical well-being. Evidence suggests that excess caregiver burden is associated with depression (22, 23), a decline in physical health, and increased healthcare utilization (24). Caregivers of older adult hip fracture patients in Singapore have been found to experience significant stress that begins as early as hospital admission and continues to remain high even six months post-admission (25). Role strain may also arise if caregiving responsibilities compete with labor market participation and compromise the caregiver's performance in either role (26). Caregivers who are employed may also find it challenging to maintain work roles while assisting a family member, as evidenced by their reported missed days, interruptions at work, leave of absence, and reduced productivity because of their caregiving obligations (27). Moreover, the competing demands of caregiving can affect the quality of caregiving, thus hindering a functional recovery in the patient.

These issues highlight the importance of enhancing functional recovery in hip fracture patients to reduce dependency and facilitate independent living within the community. Moreover, to ensure that a patient and family-centered care approach is adopted, this research aims to actively engage stakeholders (including doctors, physiotherapists, hip fracture patients, and caregivers) using the systems modeling methodology of Group Model Building (GMB) to elicit the factors that influence hip fracture recovery, incorporating a feedback perspective to inform system-wide interventions.

Most research on hip fracture recovery trajectories tends to concentrate on identifying factors associated with hip fracture recovery using models such as the growth mixture modeling approach (28) and logistic regression (29). Even with the evident contribution of these approaches, however, a new approach is needed to tackle the critical question relevant to clinical and policy action, i.e., what are the mechanisms by which these determinants of hip fracture recovery operate to promote or hinder recovery? Our study suggests how functional decline and recovery manifest through an interplay of stressors (hip fracture) and how individuals respond to those stressors (30–32). Complicating the dynamics of hip fracture trajectory of functional ability is the presence of feedback loops, in which variables are both the cause and effect (e.g., stressors reduce functional ability, and reduced functional ability promotes the occurrence of stressors). Using a qualitative system dynamics method through the engagement of stakeholders in a GMB can provide a valuable complement to empirical studies for understanding dynamically complex phenomena. Based on explicit, testable hypotheses about causal relationships, a model promotes developing and testing improved hypotheses about hip fracture recovery and identifying potential interventions to optimize recovery based on underlying drivers of functional loss or recovery.

## 2. Methods

Group Model Building is a participatory system dynamics method that engages stakeholders in developing conceptual maps and simulations for complex problems to gain a whole system perspective, leveraging the diagramming conventions of systems modeling (33, 34). The stakeholder's engagement in GMB refers to the process in which stakeholders are deeply and actively involved in model construction through the exchange, assimilation, and integration of mental models into a holistic system description (35). The GMB activities use ScriptMap (36) to describe the sequences of activities in a GMB session. Depending on the negotiated time with stakeholders, specific scripts are selected to ensure that the aim of the GMB is achieved during the session. The GMB method was used for this research to engage diverse stakeholders to develop a structured, explanatory, and coherent set of interconnected statements/theories of the factors that promote or inhibit hip fracture recovery.

### 2.1. Study context

The research team from Duke-NUS Medical School conducted two half-day workshops in Singapore on 29th August 2022 and 31st August 2022. 25 stakeholders participated in the workshop, representing Singapore General Hospital, Changi General Hospital, St. Andrew's Community Hospital, patients, caregivers, and researchers. The stakeholders include three medical doctors, six physiotherapists, two nurses, eight hip fracture patients, one caregiver, and five researchers. Informed consent was obtained from all participants before the start of the workshop sessions. The stakeholders were selected from doctors, nurses, physiotherapists, nurses, patients, and caregivers in a resilience and hip fracture recovery study funded by the National Medical Research Council of Singapore.

### 2.2. Study design

Group exercises based on two main scripts from GMB literature (37) were conducted with the stakeholders over the two half-day workshops. The outcome was to develop a preliminary qualitative model of the factors that promote or inhibit hip fracture recovery among older adults in Singapore and to identify leverage points for interventions to improve recovery. The activities were designed to build on each other to enhance understanding and participation among the stakeholders. Table 1 shows the list and sequence of activities. The first session focused on providing a comprehensive update on the demographic characteristics of the patients recruited for the ongoing hip fracture study to understand the impact of psychological resilience on hip fracture recovery and introduce the stakeholders to basic principles of systems thinking and system dynamics. The second session of activities focused on presenting a preliminary conceptual model of hip fracture recovery, eliciting stakeholder comments regarding the model structure, and clarifying the model concept, variables, and polarities. For the third session, stakeholders were asked to list all the factors that promote or inhibit hip fracture recovery not represented in the preliminary concept model. Lastly, the stakeholders were led by the facilitator (a research team member) to explore the interactions and interdependences among these factors and identify leverage points for interventions. The workshop scripts and procedures planned for the sessions have been approved by IRB.

**Table 1:** List and sequence of group activities of the GMB.

Agenda	Team Activity
Day #1	
Introduction and Background	• Introduction of the research team and stakeholders. • Background and aims of the GMB workshop.
System Dynamics Introduction	• Introduce stakeholders to causal loop diagramming. • Draw a bathtub with a faucet and drain. Use it to explain stock and flow.
Concept Model Presentation	• Presentation of concept model. • Distribution colored sheets for writing questions/comments/clarification of the concept model. • Facilitator collects the sheets and clusters on the wall and goes through them with stakeholders.
Variable elicitation	• Elicit factors: distribute colored sheets for writing factors that promote or inhibit hip fracture recovery. • In a round-robin fashion, stakeholders present factors that promote or inhibit hip fracture recovery. • Cluster the variables on the wall. • Group the variables into sub-groups.
Day #2	
Structure Elicitation	• Sketching the concept model on white paper and fixing it on the wall. • The facilitator selects a sub-group of the clustered factors, then chooses a variable at a time and asks stakeholders to identify the connection between the factor and the concept model. • Facilitators ask questions to establish the nature of the causal relationship. • After establishing the causal relationship, the facilitator asks a stakeholder to summarize by telling a story embedded in the causal relationship.
Exploration of Policy Options	• The facilitator leads the discussion with the stakeholders to identify leverage points for intervention to improve hip fracture recovery.

### 2.3. GMB exercise

A detailed description of the activities conducted during the workshop is provided herein. After introducing the agenda for the workshop, the stakeholders were divided into three groups. Each group consists of individuals from different backgrounds and organizations. The facilitators for the workshop clarified the purpose of the meeting, provided a detailed update on the patients recruited for the study, introduced stakeholders to basic principles of systems thinking and system dynamics, and described in detail the expected output from the two half-day workshops.

#### 2.3.1. Presentation of preliminary concept model

This exercise aimed to introduce the stakeholders to the initial conceptual model of functional loss and recovery from a hip fracture developed by the research team and elicit suggestions to improve the model structure. Following a stepwise and detailed explanation of the initial conceptual model, the stakeholders were allowed to review and critique the conceptual model of functional loss and recovery individually and as a group. The guiding question presented to stakeholders to facilitate discussion was: *What is wrong with this conceptual model?* Each group was given 30 min to review the conceptual model, discuss it among the group members, and list each of their questions and comments on separate post-it notes. In a round-robin fashion, each group presented one critique or clarification at a time. The process was repeated across all groups until all reviews, comments, suggestions, and clarifications were presented. Each critique, comment, suggestion, and clarification listed on post-it notes was affixed to a wall. The facilitators sought clarifications to ensure a common understanding among the stakeholders. The workshop facilitator reviewed all the post-it notes with the stakeholders to ensure that all the critiques, comments, suggestions, and clarifications were addressed to the satisfaction of all the stakeholders.

#### 2.3.2. Variable elicitation exercise

The exercise aimed to elicit causal factors that promote or inhibit hip fracture recovery. The stakeholders were urged to list all direct and indirect factors influencing hip fracture recovery based on personal and institutional experience. The guiding question provided to the stakeholders to stimulate discussion was: *What are the factors that promote or hinder hip fracture recovery, based on your experience as a patient, doctor, physiotherapist, caregiver, or nurse, that is not included in the initial conceptual model?* Like the earlier exercise, each group was given 30 min to discuss and list all the variables they believe have a role in hip fracture recovery on separate post-it notes. In a round-robin fashion, each group presented one factor at a time. The process was repeated across all groups until all factors that promote or hinder hip fracture recovery identified by each group were discussed. Each factor is listed on a post-it note and affixed to a wall after clarification from the facilitator to ensure common understanding. After that, the research team from Duke-NUS Medical School clustered all the factors listed into 14 sub-groups (see Appendix A). They are nutrition, caregivers, resilience building, comorbidities, expectation of recovery, family, care at home, communication, pain perception, income, education, individual factors, accessibility of resources, and healthcare professionals’ perspective.

#### 2.3.3. Structure elicitation exercises

This group exercise involves connecting the factors elicited by the stakeholders to the conceptual model. It began with a brief overview of the conceptual model by the facilitator. After that, the facilitator led the stakeholders to connect the factors by starting from the sub-group with the least factors. First, elements in a sub-group are randomly selected. Stakeholders are asked to suggest how that factor is connected to the conceptual model and provide a personal or institutional experiential-based story related to that factor. When another stakeholder challenges a proposed connection or relationship, the facilitator helps to clarify the concerns until a consensus is reached. The process was continued until all the elements in the 14 sub-groups were used. The facilitator discarded factors that became redundant based on the advice of the stakeholders. In connecting all the factors, initially suggested connections were revisited and changed when necessary to represent the complex interconnections between the elements better. Additional variables subsequently identified were also added to represent the causal relationships between variables better. Details of exercise are reported in the results. The field model developed with stakeholders can be found in Appendix B.

## 3. Results

**Figure 1:**
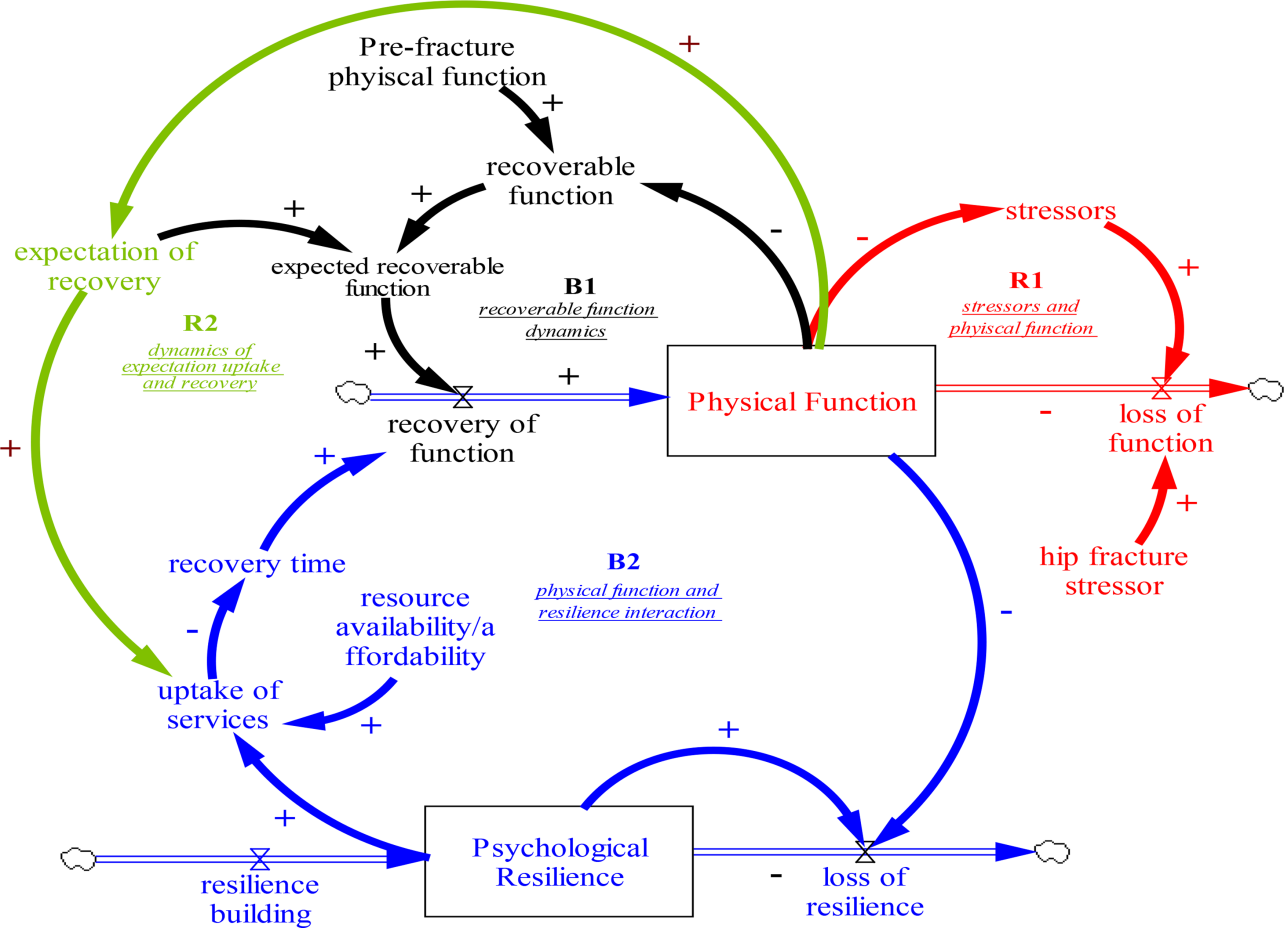
Base model on the stressor-induced loss of physical function and recovery.

**Figure 2:**
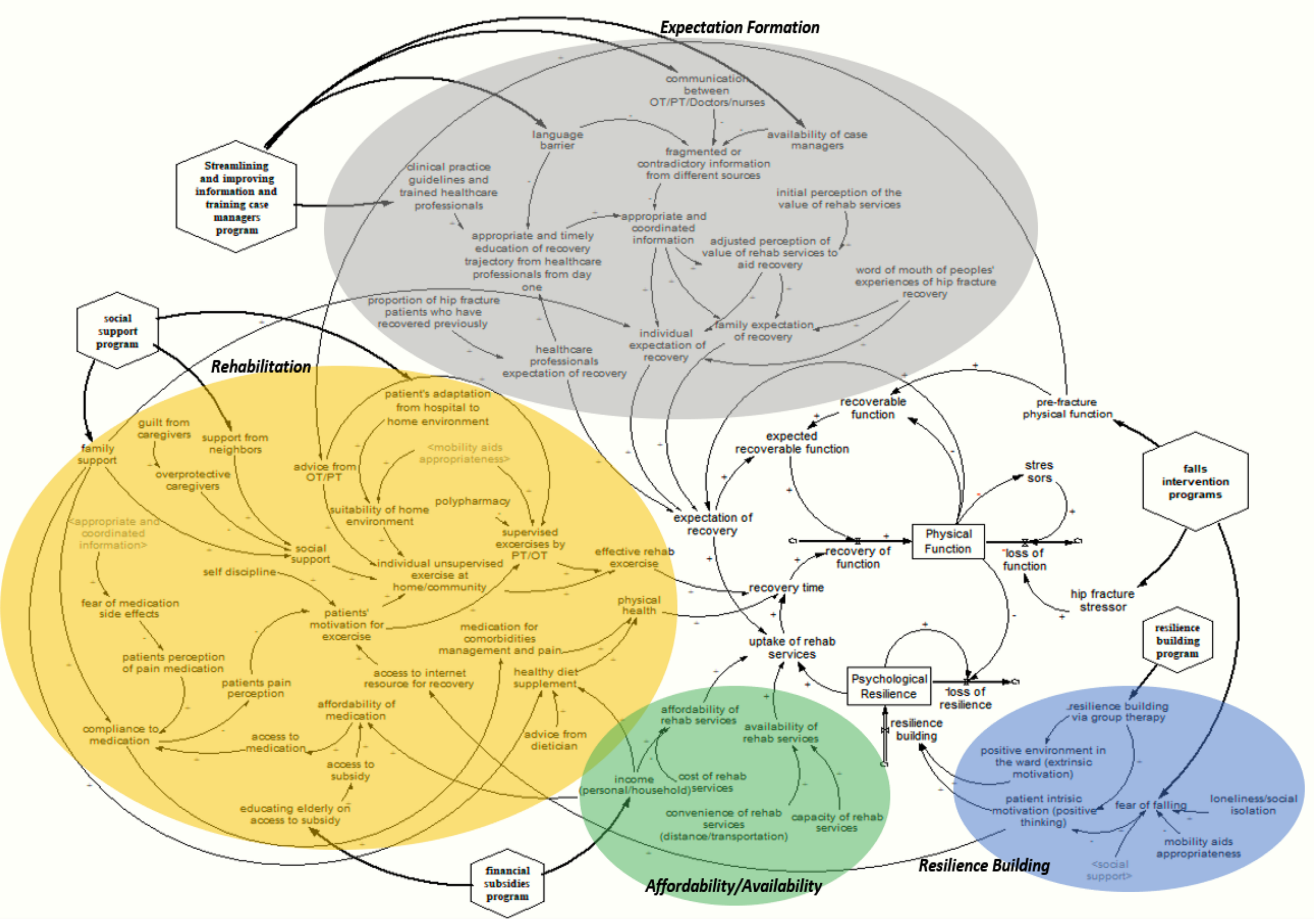
Qualitative model developed with stakeholders.

Results: This section describes the preliminary concept model (Figure 1) and the qualitative model developed with stakeholders (Figure 2). The suggested interventions to improve hip fracture recovery is presented in the discussion. The qualitative model developed with stakeholders was divided into four segments, as shown in Figure 2.

### 3.1. Preliminary concept model

Within the context of the conceptual model (see Figure 1) adapted from the reference as cited (38) as part of the study by the authors, we define the key variables in the model as follows. Physical function refers to the level of activity an individual achieves in his day-to-day function. This function comprises cognitive and physical capacity that influence an individual's ability to perform activities of daily living (39–41). Pre-Fracture physical function is the pre-fracture function in the absence of stressors. It is the reference function, which is assumed to remain unchanged over 12 months in the context of this study. It provides a benchmark for comparing physical function. A stressor is any event (e.g., hip fracture) that reduces functional ability. Larger stressors correspond to larger reductions in physical function. Psychological resilience reflects the psychological features of an individual that promote or inhibit recovery. A person with high psychological resilience will respond to stressors in a manner that promotes rapid recovery from physical function loss. In contrast, a person with low psychological resilience will respond in a way that leads to a slow recovery or deterioration of physical function (42–44). The expectation of recovery is what an individual expects may recover from the difference between pre-fracture physical function and physical function. This expectation could be a reflection of an individual's optimism (45). Individuals with a higher expectation of recovery should have a complete recovery, while individuals with lower expectations will likely have less complete recoveries. Despite other definitions of resilience that encompass some expectations, we define the expectation of recovery as a distinct characteristic from our definition of resilience. The feedback loop R1 describes the feedback relationship between stressors and physical function. R1 stipulates that individuals with low physical function are more likely to experience stressors than those with higher physical function. This increased rate of stressors exacerbates the reduction in physical function, ultimately leading to a greater vulnerability to future stressors. These causal relationships form a reinforcing feedback loop where exposure to stressors decreases physical function, leading to a higher likelihood of experiencing more stressors over time. Feedback loop B1 describes the balancing feedback relationship between recoverable loss of function and recovery rate. Individuals with a higher expectation of recovery will expect a larger recoverable function lost due to stressors. The loss of actual function due to stressors creates a gap between pre-fracture physical function and physical function. The gap is referred to as “recoverable function,” a weighted average difference between the pre-fracture physical function and physical function. The weight is the value assigned to the “expectation of recovery” and can vary between individuals and within individuals over time. A weight of one indicates an expectation that the potential for the physical function is equivalent to the expected function. In contrast, a weight of less than one means a lower recovery expectation. When an individual has a higher expectation of recovery (close to one), this individual will engage more in activities that promote the recovery of that lost function. Hence, the individual will recover more quickly and close the gap between the individual's pre-fracture physical function and current physical function. As that gap closes, the rate at which the individual recovers decreases until the gap closes. Feedback loop B2 describes the negative reinforcing relationship between physical function and psychological resilience. When an individual experiences a decline in physical function due to stressors, the individual will not be able to engage in activities that maintain the psychological, social, and physiological drivers of recovery. Hence, an individual with poor physical function also experiences a more rapid depletion of psychological resilience. This individual will be less likely to take up services and engage in other activities that promote recovery of the lost function. Therefore, the individual will have a longer recovery time and is less able to improve his physical function. Feedback loop R2 relates the dynamic relationship between the expectation of recovery, uptake of services, and physical function. We postulate here that high expectations of recovery will stimulate the appropriate use of services to promote recovery of physical function. Consequently, as physical function recovers expectation of recovery is likely to increase further.

## 4. Qualitative model developed with stakeholders

This section describes the expanded qualitative conceptual model developed with key stakeholders. The expanded qualitative model is divided into four sectors: (a) expectation formation, (b) rehabilitation, (c) affordability/availability, and (d) resilience building.

### 4.1. Expectation formation

Figure 2 shows the qualitative model developed with stakeholders, clearly indicating the expectation formation sector. Stakeholders identified three main factors that contributed directly to the “expectation of recovery” of hip fracture patients. They are (a) individual expectation of recovery, (b) family expectation of recovery, and (c) healthcare professionals' expectation of recovery.

Stakeholders suggested that individual expectation of recovery was driven by four main factors: (a) the appropriate and coordinated information provided to the patients while at the hospital, (b) perception of the value of rehabilitation services, (c) word of mouth of other patients' experiences of hip fracture recovery, and (d) family support. Appropriate and coordinated information provided to the patients is determined by the factually correct and timely education on hip fracture recovery. This education should be delivered by healthcare professionals immediately upon a patient's admission to the hospital. *A healthcare professional argued that the information provided to patients must also be tailored to the specific needs of the patient*. When information is provided to patients in a coordinated manner, patients' perception of the value of rehabilitation services for their hip fracture recovery is substantially improved. In contrast, incoherent and uncoordinated provision of information is likely to worsen a patient's negative perception of the value of rehabilitation services. Patient stakeholders felt that fragmented and contradictory information was often communicated to them during their stay in the hospital. Furthermore, *A healthcare professional stakeholder mentioned that the negative tone of some healthcare professionals might negatively affect a patient's perception of the recovery process, for instance, by only highlighting the high risk of surgery without talking about the expected recovery trajectory*. *Hence*, stakeholders suggest that having clear clinical practice guidelines to guide healthcare professionals in engaging patients and family members will likely enhance the recovery trajectory of patients. Likewise, the stakeholders postulated that fragmented and contradictory information from different sources could also be caused by language barriers, lack of availability of care managers, and poor communication between healthcare professionals and patients. Patients in our stakeholder group agreed with such a sentiment. One stakeholder *mentioned that during her time at the hospital, she had to assist the healthcare team to translate their care instructions for a fellow patient in the ward because the healthcare team did not speak the Chinese dialect with which the patient was most comfortable communicating in.* Another factor influencing an individual's expectation of recovery is the perception of the value of rehabilitation services. This perception is postulated to be shaped by an initial perception of the value of rehabilitation services held by patients and the information provided to patients regarding the role of rehabilitation services and patients' recovery. Stakeholders also felt that word of mouth from other patients' experiences of hip fracture recovery would influence their expectations of recovery. This word of mouth consists of stories about other patients' experiences of hip fracture recovery. *A doctor stakeholder shared that he uses a positive story about a previous patient's experience as a way to foster a positive expectation of recovery*. These positive recovery stories can potentially increase an individual's expectation of recovery, whereas negative recovery stories are likely to lower individuals' expectations of recovery.

Stakeholders suggested that better family support, especially from caregivers involved in a patient's recovery, is associated with the positive individual expectation of recovery. The family's expectation of recovery was found to have similar influences as the individual's expectation of recovery, and they are (a) appropriate and coordinated information, (b) perception of the value of rehabilitation services to aid recovery, and (c) word of mouth of other patients’ experiences of hip fracture recovery. As suggested by the stakeholders, appropriate and coordinated information delivered to the family regarding the patient's recovery trajectory will directly influence the family's expectation of recovery and the family's perception of the value of rehabilitation services to aid recovery. *Stakeholders believed that when family members positively perceive the value of rehabilitation services, they will support the patient through their recovery journey with their time, financial resources, and rehabilitation equipment and provide encouragement, amongst other means and resources, to aid their functional recovery in the long run*. Thus, the appropriate and coordinated information received by the family, the positive perception of the value of rehabilitation services to aid in recovery, adjusted by known stories of hip fracture recovery of other individuals, determine the family's expectation of recovery.

Lastly, stakeholders shared that healthcare professionals have their own expectations of patient recovery. This is influenced by their institutional and personal knowledge and experiences working with hip fracture patients. It is hypothesized that healthcare professionals adjust their advice to patients and family members on a patient's recovery trajectory based on their expectation of recovery informed by past experiences in treating similar patients.

### 4.2. Rehabilitation

The stakeholders identified two main factors related to rehabilitation that directly contributes to recovery time. The factors are effective rehabilitation exercises and physical health. Based on stakeholder knowledge, effective rehabilitation exercises were hypothesized to be driven by patients' participation in supervised exercises provided by physiotherapists or occupational therapists (PT/OT) and unsupervised exercises conducted at home or in the community by the patient. Supervised exercise with the PT/OT is determined by the uptake rate of supervised exercise referral by PT/OT (advice from PT/OT) and adjusted by patients' motivation to exercise to improve recovery from hip fracture, availability of appropriate mobility aids to support patients, and polypharmacy. According to the Stakeholders, mobility aids help the gradual progression of exercises tailored to the patient's recovery. Physiotherapists in the stakeholder group assert that PT/OT advice involves making recommendations of individualized exercises tailored to the patient based on the knowledge of the patient's pre-fracture physical function*. A physiotherapist gave an example of when a patient*'*s recovery journey begins; they first do exercises with a walking frame, then do more advanced exercises. They suggested that it was important for the physiotherapist to start by teaching the simple patient tasks first before gradually moving on to more complex tasks*. Thus, advice from PT/OT is influenced mainly by patients' pre-fracture physical function. These recommendations are prescribed to patients during supervised physiotherapy appointments. The stakeholders postulate that the following factors influence the uptake of individual unsupervised exercise at home. They are the suitability of the home environment, social support, advice from PT/OT, and patients' motivation to exercise. The suitability of the home environment is hypothesized to be influenced by patients' adaptation from the hospital to the home environment and the availability of appropriate mobility aid. It was argued that patients who engaged in individual unsupervised exercise at home are primarily individuals who have managed to adapt to the home environment following their discharge from the hospital. A suitable home environment enables patients to practice exercises in a comfortable and familiar space. *A doctor stakeholder suggested that not everyone is interested in going for rehabilitation sessions but may prefer to do their exercises at home where they feel safe and more comfortable*. In addition, social support was argued to be a crucial factor for hip fracture patients to engage in individual unsupervised exercise at home. Social support comes from the support shown by family members and neighbors. However, overprotective caregivers can negatively affect the level of social support a hip fracture patient experiences. The stakeholders emphasized that the higher the caregiver overprotectiveness (especially in Singapore, where most hip fracture patients are likely to have lived-in foreign domestic workers as caregivers), the greater likelihood that the hip fracture patients will not engage in individual unsupervised exercise to aid recovery. *Patients in the stakeholder group shared that overprotective caregivers (including family members) may prematurely terminate rehabilitation sessions or reduce them significantly because of pain and discomfort experienced by the patient*. *Furthermore,* overprotective caregivers may be excessively supportive and remove patients' independence due to guilt and fears of a coming fall.

Patients' motivations for exercise are influenced by self-discipline, patients' pain perception, and access to internet resources for recovery. *Patients in the stakeholder group agreed that self-discipline is essential for motivating oneself to exercise, despite the presence of fear of falling after a hip fracture*. Self-discipline is an individual characteristic, and stakeholders believe it is crucial for continuous exercise. Another factor that affects patients' motivations for exercise is a patient's pain perception. A patient's pain perception was argued to be influenced by compliance with medication which is determined by access to medication, patients' perception of pain medication, and family support. Increased perceived pain and discomfort would lead to less willingness to exercise. A *nurse in the stakeholder group shared that patients' negative views of medication affect compliance to take the appropriate prescribed dose of medication*. Access to medication required by hip fracture patients is a function of affordability determined by personal/household income and access to medication subsidies provided by the government. *According to the stakeholders, subsidies are available to help patients afford the required medication. However, many patients are often unaware that these subsidies are available or lack the knowledge to navigate the bureaucracy to apply for them*. Therefore, by educating patients on public subsidies the affordability and accessibility to medication. Another factor influencing patients' pain perception is the fear of medication side effects. *Stakeholders argued that patients have a negative perception of pain medication due to misconceptions about the potential side effects of medication and fears of becoming over-reliant on pain medication*. Healthcare professionals can address these fears by providing appropriate and coordinated information to patients at the hospital. Hence, the clinician team's appropriate and coordinated information would be vital to clarifying the misconceptions, providing assurance, and alleviating concerns. Lastly, accessing internet resources regarding recovery may help educate patients on the value of exercise, increasing patients' motivation for exercise. Patients who actively access resources independently are influenced by their intrinsic motivation for recovery.

On physical health*,* factors such as (a) medication for comorbidities and pain management and (b) healthy diet and supplementation were identified as the main drivers. Patients' compliance with medication for managing comorbidities and pain affects patients' physical health. As explained earlier, compliance with medication can be affected by the access and affordability of medication, as well as patients' perceptions of pain medication. Furthermore, a healthy diet and optimal nutrition would also directly impact physical health. Dieticians advise healthy dietary habits and optimal nutrition in the hospital post-hip fracture surgery. *Patients in the stakeholder group also argue that healthy diets may be more expensive. Thus, they feel that patients need sufficient financial resources to support these changes to their eating habits*. In addition, family support, through consistent reminders or preparation of nutritious food, is vital in ensuring patients adhere to their new healthy diets.

### 4.3. Affordability/availability of rehabilitation services

Stakeholders concurred that the uptake of rehabilitation services is directly affected by two main factors: (a) affordability and (b) the availability of rehabilitation services. The affordability of rehabilitation services is determined by personal/household income and the cost of rehabilitation services. One's income includes personal income and/or the income of other household members. *Many stakeholders agreed that immediate family members might be able to financially support hip fracture patients on their recovery journey, contributing to greater uptake of rehabilitation services. Stakeholders highlight that most hip fracture patients are usually unable to work and sustain a regular income after an operation*. Therefore, having household members who can assist in paying for services could mitigate income loss and encourage patients to take up rehabilitation services. On the contrary, if the cost of rehabilitation services is too high or unaffordable, patients would be less likely to take up any rehabilitation services.

The availability of rehabilitation services also plays a significant role in the uptake of services. The availability of rehabilitation services is directly affected by (a) the convenience of rehabilitation services and (b) the capacity of rehabilitation services in the healthcare system. *Patients in the stakeholder group emphasized that easily accessible rehabilitation services in the community where hip fracture patients live are needed*. As recovering hip fracture patients have poor mobility, they are unwilling to travel to a rehabilitation center, deterring them from utilizing these services. However, should a rehabilitation center be located nearby and easily accessible by transport, this would increase the likelihood of a patient using the rehabilitation services. The overall capacity of rehabilitation services is essential in the uptake of services. *Stakeholders mentioned that even if they were willing to take up services, they would only be able to access them if high-quality services were available*. This means that the provision of good quality services, in addition to convenience and availability, can also be a reinforcing mechanism in encouraging patients to attend rehabilitation on a sustained basis.

### 4.4. Resilience building

Stakeholders emphasized how resilience building is shaped by two factors (a) a person's intrinsic motivation, described by many stakeholders as having “positive thinking, and (b) a positive environment or extrinsic motivation. Intrinsic motivation, combined with an encouraging extrinsic environment, develops and strengthens psychological resilience. Meanwhile, a positive environment starts from the ward and continues into the community*. One patient in the stakeholder group shared about a church outreach group that reaches out to older adults living alone to do regular exercises together*. Stakeholders discussed how a patient”s intrinsic motivation, or the presence of positive thinking, is shaped by patients” fear of falling. Fear of falling, in turn, is affected by three factors, (a) loneliness/social isolation, (b) social support, and (c) appropriate mobility aid. Stakeholders mentioned how loneliness and/or social isolation, coupled with relatively poor social support, can lead to an increased fear of falling. As discussed earlier, social support consists of support from neighbors and family members. Their regular presence around the patient helps the patient to recover psychologically. In addition, using appropriate mobility aids can reduce the patient's fear of falling by empowering them to move independently and confidently.

A hip fracture patient's intrinsic motivation and a positive environment can be facilitated by participating in group therapy. Stakeholders indicated how group therapy could build resilience by providing a platform for mutual guidance and encouragement*. A physiotherapist in the stakeholder group mentioned that patients share information and learn from one another when they exercise together during group therapy*. Through group therapy, patients can share exercise-related information and learn exercise techniques from one another, including how others conduct their exercises, adjusting their techniques, and motivating each other. Group therapy also provides a platform for patients to connect with others who share similar experiences, serving as an avenue for social interaction and companionship. Patients tend to be more motivated to continue their therapy if they participate in a group rather than on their own. *As shared by stakeholders, group therapy enables one to be aware of the ordinary, everyday shared experiences and challenges other hip fracture patients face*.

## 5. Discussion

First, the insight that recovery of loss of function due to hip fracture is attributed to (a) the recognition of a gap between pre-fracture physical function and current physical function; and (b) the marshaling of psychological resilience to respond promptly to a physical functional loss via uptake of rehabilitation services is supported by findings from studies as cited (46–49) and has several policy implications. The recognition of the gap between current and pre-fracture physical function and marshaling psychological resilience to close this gap was found to be determined by the expectation of recovery, which is in turn influenced by the quality of the information provided to patients and their families by healthcare professionals and the appropriate and coordinated delivery of this information. From a policy perspective, this suggests that hip fracture care must prioritize patient education and coordinated communication to raise the expectation of recovery. A rise in the expectation of recovery will lead to increased uptake of rehabilitation services and, consequently, better recovery outcomes. Thus, it is essential to emphasize that well-coordinated information provided by healthcare professionals to patients is a critical component for patients' hip fracture recovery journey. This insight informed the identification of a program intervention by the stakeholders that focuses on streamlining and improving the information provided by the healthcare professional to patients and family members and ensuring that the information and the delivery process are carefully detailed in clinical practice guidelines. This information includes early goal setting (setting realistic expectations and aligning family and patients' expectations), laying out the appropriate rehabilitation services needed, providing dietary recommendations, having open communication between patients, families, and healthcare professionals, and adjudicating differences in expectations. This involves clinicians managing expectations and incorporating preferences, needs, and decisions from patients and their families instead of dictating the course of recovery via a top-down approach. A shared understanding of what is expected during recovery is essential for all stakeholders so as to facilitate better recovery outcomes.

In addition, healthcare professionals can provide caregivers of patients with systematic training and specific guidelines, such as concise, appropriate, and targeted information to support patient recovery at home. Stakeholders suggested the need to leverage IT platforms to coordinate information sharing to help the healthcare team stay on the same page when communicating with patients. More importantly, healthcare professionals required to deliver the information to patients should be well-trained and familiar with what they must provide. To prevent potential miscommunication or dissemination of fragmented information, stakeholders suggested the need to develop a program that aims to train case managers that specialize in coordinating information provided by healthcare professionals. The role of these case managers ensures that there is one reliable contact point between healthcare providers and hip fracture patients and their families. This approach ensures that patients and their families have a clear and comprehensive understanding of the aligned expected recovery trajectory and the expected actions of the patients and families to support the recovery process. Stakeholders observed that case managers who were readily available to answer questions from patients and family members helped to build a more apparent, more explicit expectation of recovery after a hip fracture.

Second, the findings from this study suggest that support from family caregivers, friends, and neighbors is essential for the uptake of effective rehabilitation services. This has several policy intervention implications. Social support refers to providing emotional support, allowing hip fracture patients to engage in prescribed exercises recommended by physiotherapists, helping patients use mobility aids to walk, creating a positive environment that encourages patients to adhere to treatment and recovery regimes, and decreasing fear of falling. This insight led the stakeholders to identify social support programs that educate family members, caregivers, and the public about the importance of social support to older individuals in dealing with life stressors, including hip fractures. Nevertheless, it is vital to emphasize that overprotective support from caregivers (family or neighbors) can inhibit patients' recovery within the social support mechanism, as overprotective caregivers may actively limit patients' engagement with rehabilitation activities. Hence, healthcare professionals need to guide patients' recovery process by continuously engaging caregivers to ensure that they understand and are engaged with the expected trajectory of recovery and the expected actions they should be undertaking to promote recovery.

Third, the insight that hip fracture patients need financial support to subsidize the cost of medication, rehabilitation services, and mobility aids was reinforced by stakeholders' experience of the significant cost burden of hip fracture. Stakeholders indicated that the process of recovery from hip fractures is costly and requires substantial resources. For most older adults, the financial burden, especially if they have no external support from children, family members, and the government, is significant and can affect their ability to take up essential services and negatively impact their recovery trajectory. This finding informed the stakeholder's identification of a financial subsidy program that provides subsidies to support hip fracture patients, especially low and middle-income patients, who require financial assistance to enhance rehabilitation services uptake and promote recovery. Furthermore, it is also essential to educate patients and their caregivers on the subsidies available and how to apply for them to increase their access to subsidies.

Lastly, the insight that in the wake of a stressor-induced physical function loss (such as a hip fracture), a person with high psychological resilience will respond in a way that promotes rapid recovery, while an individual with low psychological resilience will react in a way that leads to a slow recovery or deterioration has policy/intervention implications. Based on this insight, stakeholders suggested that healthcare systems should consider implementing resilience-building programs that help older adults to build psychological resilience to prepare them for responding to life stressors over their life course. Psychological resilience assists individuals in developing individual characteristics and the ability to cope with life stressors. The findings indicate that building psychological resilience among patients includes engaging them through a mix of formal and informal support. Formal support structures include group therapy in hospital settings, hip fracture patient group creation, and community support. Informal support includes social support from the patient's family, friends, and community. Creating a positive-eco system for patient recovery is pivotal to building psychological resilience for better recovery outcomes. Moreover, empowering patients through elements of patient-centered care (i.e., alignment and management of patient expectations, providing coordinated information and care through case managers) can also increase patients' self-efficacy and strengthen their resilience toward recovery. The stakeholders also suggested that the psychological resilience programs should be paired with fall prevention programs. According to the stakeholders, targeted fall intervention programs could help improve the overall pre-fracture physical function of the general population. A falls prevention program aims to reduce the frequency of falls by building strength and addressing associated risks and fears related to falls. A fall is a significant stressor for older adults and a large contributor to the initial hip fracture; hence it could significantly impact their life course trajectory. Therefore, a comprehensive fall prevention program that includes components of resilience building can reduce the likelihood of falls and contribute to faster recovery from hip fractures.

### 5.1. Strengths and limitations

The strength of the present study is that it is one of the first to delve into the recovery processes of hip fracture patients, incorporating a multidisciplinary set of stakeholders who are directly affected or are involved in the care of hip fracture patients. It is also one of the first to synthesize the responses of occupationally diverse and multi-ethnic stakeholders into a comprehensive and extensive dynamic model.

A limitation of this study is that this study is conducted in Singapore, and therefore, the model may be applicable to a different context. The responses from participants in this study are based on local experience with a specific healthcare configuration that may operate differently in another country. Likewise, as the older age groups change in terms of health literacy and other factors, changes may be needed to be made to the model. The study did not actively discuss the hip fracture journey of individuals with dementia. Future studies should consider including the needs of hip fracture patients with dementia.

## 6. Conclusion

This paper explains the factors that promote or inhibit hip fracture recovery, incorporating a feedback perspective to inform system-wide interventions. The insight from the study suggests that recovery of loss of function due to hip fracture is attributed to (a) the recognition of a gap between pre-fracture physical function and current physical function; and (b) the marshaling of psychological resilience to respond promptly to a physical functional loss via uptake of rehabilitation services. However, recognizing the gap and marshaling psychological resilience to close it is determined by the expectation of recovery, which is influenced by the quality of the information provided to patients and their families by healthcare professionals and the appropriate and coordinated delivery of this information.

In light of these insights, hip fracture care in Singapore should consider streamlining and improving the information to patients and family members and ensuring that the information and the delivery process are carefully detailed in clinical practice guidelines. The streamlining of the information has the potential for the healthcare team and caregivers to reinforce the positive benefits of rehabilitation to patients to improve recovery expectations. In addition, policies that focus on providing social support to hip fracture patients, financial subsidies, and resilience-building programs have the potential to facilitate hip fracture recovery among older adults in Singapore.

## Data Availability

The original contributions presented in the study are included in the article, further inquiries can be directed to the corresponding author.

## Appendix

Appendix A: Factor elicitation variables

Appendix B: Qualitative model developed with stakeholders.
